# X-Linked Retinoschisis in Juveniles: Follow-Up by Optical Coherence Tomography

**DOI:** 10.1155/2017/1704623

**Published:** 2017-02-14

**Authors:** Qin-rui Hu, Lv-zhen Huang, Xiao-li Chen, Hui-ka Xia, Tian-qi Li, Xiao-xin Li

**Affiliations:** Department of Ophthalmology, Peking University People's Hospital, Key Laboratory of Vision Loss and Restoration, Ministry of Education, Beijing Key Laboratory for the Diagnosis and Treatment of Retinal and Choroid Diseases, Beijing, China

## Abstract

*Purpose.* To explore the structural progression of X-linked retinoschisis (XLRS) in patients by using spectral-domain optical coherence tomography (SD-OCT).* Design.* Retrospective, observational study.* Methods.* Patients who were diagnosed with XLRS by genetic testing underwent comprehensive ophthalmological examinations from December 2014 to October 2016. Each eye was measured by SD-OCT using the same clinical protocol. A correlation between best-corrected visual acuity (VA) and SD-OCT measurements was observed.* Results.* Six patients demonstrated retinoschisis (12 eyes) and typical foveal cyst-like cavities (10 eyes) on SD-OCT images with a mean logMAR VA of 0.48. The median age was 7.5 years at the initial visit. Their foveal retinal thickness (516.9 *μ*m) and choroid thickness (351.4 *μ*m) decreased at a rate of 38.1 and 7.5 *μ*m, respectively, at the 10.5-month follow-up visit; however, there were no significant differences (*P* = 0.622 and *P* = 0.406, resp.). There was no significant correlation between VA, the foveal retinal thickness, and subfoveal choroid thickness.* Conclusions.* SD-OCT images for XLRS patients during the juvenile period revealed no significant changes in the fundus structure, including the foveal retinal thickness and choroid thickness within one-year follow-up. There was a lack of correlation between VA, foveal retinal thickness, and subfoveal choroid thickness.

## 1. Introduction

X-linked retinoschisis (XLRS) is an inherited vitreoretinal dystrophy and is characterized by foveal schisis in patients [1–3]. The prevalence of XLRS varies from 1 : 5000 to 1 : 25000 [2, 4–6]. OCT is a safe and noninvasive procedure to diagnose and monitor patients with XLRS [7]. Although the macular anatomy has been well studied using OCT, the follow-up and quantification of foveal retinal thickness and choroid thickness in XLRS patients have not been well documented [8].

The goal of this study was to use SD-OCT to evaluate the progression of structural changes in the retina in XLRS patients and their correlation with visual function during the follow-up period.

## 2. Methods

The study procedures were performed in accordance with institutional guidelines and the Declaration of Helsinki. Informed consent was obtained from all patients after a full explanation of the procedures.

All patients were diagnosed with X-linked retinoschisis by genetic testing. The following data about patients were collected: funduscopic examination data, measurements of visual acuity (VA) with a standard logMAR visual acuity chart, and SD-OCT examination data. The images were acquired using a Cirrus HD-OCT unit (Cirrus HD-OCT; Carl Zeiss Meditec) with line scan (6 mm on the retina), a wavelength of 840 nm, and an axial resolution of 5 *μ*m. Multiple measures were obtained for the foveal retinal and subfoveal choroid thicknesses. Clinical therapies were recorded during the visit.

A paired Student's *t*-test was performed to compare paired clinical data for foveal retinal and subfoveal choroidal thickness measurements for the subjects at the first and last examination (SPSS 16.0). A Pearson test was used to correlate VA with the foveal retinal thickness and the choroid thickness. A *P* value of 0.05 was considered statistically significant.

## 3. Results

The clinical characteristics are summarized in Table 1 from December 2014 to October 2016. Eight male patients were enrolled in the study. Two patients were excluded because of vitreous hemorrhage and peripheral retinal detachment by trauma. Therapeutic scleral buckling procedures were performed for these patients. The median age of the 6 patients at the first visit was 7.5 years, with a range from 5 years to 11 years. The average visual acuity was 0.48 ± 0.21 (median: 0.45) at the first visit, whereas at the last visit it was 0.46 ± 0.28 (median: 0.35) after an average of 10.5-month follow-up period. The median foveal retinal thickness at the first visit was 566.5 *μ*m (the last visit: 454.5 *μ*m) ranging from 147.0 *μ*m to 869.0 *μ*m. The median subfoveal choroid thicknesses were 343.5 *μ*m and 351.5 *μ*m at the second visit. Of the 6 patients, there were typical foveal cyst-like cavities in 10 eyes (83.3%). Schisis in the INL was observed in all 12 eyes (100%). GCL schisis was observed frequently (83.3%) in 10 eyes, slightly higher than outer plexiform layer (OPL) schisis observed in 8 eyes (66.7%).

The average foveal retinal thickness (516.9 *μ*m) and subfoveal choroid thickness (351.4 *μ*m) decreased at a rate of 38.1 and 7.5 *μ*m, respectively, during the follow-up period, but there were no significant differences between the two visits (foveal retinal thickness, *P* = 0.622; subfoveal choroid thickness, *P* = 0.406). There was a lack of correlation between VA, foveal retinal thickness, and subfoveal choroid thickness (Table 2).

## 4. Discussion

In our study, VA remained stable during the follow-up period and did not correlate with either foveal retinal thickness or choroid thickness in XLRS patients, except for two patients who lost their vison because of vitreous hemorrhage and retinal detachment. These findings are consistent with previous reports that visual acuity is stable if no secondary event occurs [2, 9].

Our study found that both the inner and outer retinal structures of XLRS patients were affected. SD-OCT showed that the INL (100%, 12 eyes) was the most prevalent area of schisis or defect. The GCL was also frequently affected (83.3%). These results are supported by previous pathology based studies, which demonstrated that inner retinal abnormalities were the main manifestation of XLRS [10, 11].

The average foveal retinal thickness was 516.9 *μ*m and decreased to 478.8 *μ*m in our study. Foveal retinal thickness remained relatively stable during the follow-up period. The foveal cyst-like lesions underwent visible reduction only in a few cases, but the tiny cystic change was hard to clearly define and quantify, because a completely consistent baseline SD-OCT scan was nearly impossible. A previous study described progressive changes in foveal thickness and that foveal thickness was reduced to below normal over time; therefore, macular cystic-like lesions would no longer be apparent and atrophic-appearing lesions could be observed [4, 12]. Middle-aged and older patients often presented a nonspecific atrophic appearance of macular lesions [1, 9]. The present study suggests that the retina remains structurally stable in adolescence and atrophy may appear for decades. Monitoring macular change is feasible once a year using SD-OCT.

In the study by Yang et al., the average subfoveal choroidal thicknesses were approximately 358.0 *μ*m and 35 *μ*m thicker in the patient group than in the normal control group, but it failed to achieve statistical significance (*P* = 0.084) [13]. In our study, the average subfoveal choroidal thickness was 351.4 *μ*m, which was similar to the study above. Moreover, at the end of nearly one-year follow-up, the subfoveal choroidal thickness was minimally decreased by 7.5 *μ*m (*P* = 0.406). Thus, we conclude that the subfoveal choroidal thickness remains constant in adolescent XLRS patients.

**Figure 1:**
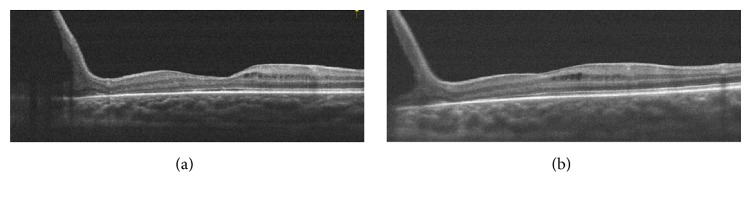
Optical coherence tomographic images for one patient. (a) Vertical images revealed a relatively normal foveal retinal thickness with peripheral retinoschisis of the fundus. (b) Image of the same eye 1.5 years later. The fundus structure did not have obvious changes.

**Figure 2:**
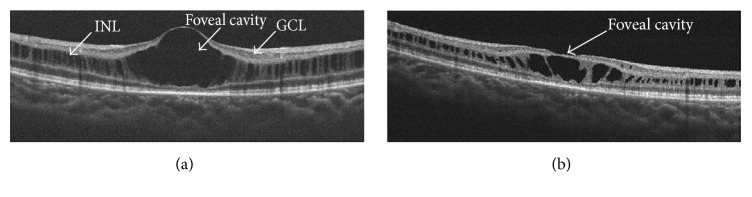
Optical coherence tomographic images for another patient. (a) Vertical images revealed defects in the ganglion cell layer (GCL) and inner nuclear layer (INL). (b) Image of the same eye 1 year later. Concave deformations appeared on the surface of the foveal cystic cavity.

However, XLRS is characterized by a high degree of clinical variability between individuals. In our study, one patient (Figure 1) showed a relatively normal SD-OCT appearance in the macular area, whereas the main pathological changes existed in the peripheral fundus. In this case, SD-OCT only provided a subtle diagnostic clue, which was not sufficient to monitor the condition. More evidence was necessary to make the diagnosis, including flash-electroretinogram (ERG) and genetic testing. For peripheral retinoschisis, a wide-field SD-OCT imaging technique or ERG may be good auxiliary methods to monitor progress. According to a previous study, wide-field SD-OCT allowed the simultaneous visualization of the macular and extra macular regions, necessary for understanding complex retinal anatomy with diffuse or multifocal schisis involving multiple retinal layers [8]. Wide-field SD-OCT scans may be a promising tool in XLRS clinical trials. For another patient (Figure 2), the SD-OCT showed a typical appearance of foveal cystoid spaces in the binocular. However, his ERG results were normal in both eyes with both a- and b-wave amplitudes within the normal range. On the SD-OCT of the fovea, although the center foveal convexity dropped over time to a slight concave profile, the peripheral retina change was not significant. In these cases, a single measurement with SD-OCT was not sufficient to evaluate the progress of the fundus structure and eye function; therefore, integrated assessments are recommended.

Choriocapillaris provides the necessary nutrients for the outer retina. However, the actual relationship and order of degeneration in the choroidal and retina interface in certain diseases remain not fully understood [14, 15]. A previous study found that choriocapillaris breakdown preceded retinal degeneration in age-related macular degeneration [16]. Data indicated that choriocapillaris breakdown occurred during normal aging and preceded degeneration of the retinal pigment epithelium (RPE) and retina. In the present study, we identified a correlation between the structure and function in juveniles. However, no correlation was found between the foveal retinal thickness, the choroidal thickness, and the visual acuity. We propose that this result is related to continuing ocular axial length growth in the sample of children with XLRS. Thirteen is considered the age marking the end of growth of the eye axial length [2, 17, 18]. In an older patient group (>13 years) in a study of XLRS by Vincent A, the refractive error was significantly more hypermetropic and the axial length was significantly shorter than the normal adult group [18]. The overall clinical picture may resemble atrophic macular degeneration in older individuals [19]. In our study, the patients were at a stage in which the general structure of the fovea and subfoveal choroid would not have dramatic atrophic changes. Slight changes of the subfoveal choroid thickness show no significant correlation with damage to vison function and obvious transformation may not be present for decades. This result was also consistent with previous studies [9, 13]. Although some subtle changes could be found, the short-term follow-up of less than one year would be unlikely to detect obvious changes in the natural course of XLRS. Particular attention should be given to the investigation of different stages for XLRS patients who may reveal a possible correlation between the retina, choroidal, and eye function.

Two patients lost their vision because of vitreous hemorrhage caused by trauma. A similar outcome was also noted by a previous study [2]. As vitreous hemorrhage is mainly caused by the rupture of unsupported blood vessels or preretinal neovascularization [19, 20], avoiding intense activity should be advised for XLRS patients.

There are several limitations of this study. It is possible that the present results do not reflect the exact changes in measurements, because a very small number of cases were recruited in this study. The patients in the present study were followed up for a short time within one year, whereas the condition remained clinically stable in the younger age group. Moreover, it is difficult to obtain consistent measurements on the SD-OCT scan at different time points when there is significant distortion of the normal anatomy. Therefore, measurements with the SD-OCT may be prone to bias.

In conclusion, SD-OCT may be used to monitor structural changes over time in patients with XLRS, but further evidence is required to ascertain any biomarkers of disease progression. In the present study, we found that schisis occurred most frequently at the INL and GCL. No obvious structural changes were observed during the follow-up period. XLRS complications are variable in the clinic, and trauma should be avoided, particularly in the adolescent. Timely and targeted measures should be considered when managing complications to prevent progressive visual deterioration and improve the visual prognosis.

## Figures and Tables

**Table 1:** Characteristics of the patients.

Eye	Age (year)	First visit			Last visit				Foveal retinoschisis			
		Foveal thickness (*μ*m)	Choroid thickness (*μ*m)	Visual acuity	Foveal thickness (*μ*m)	Choroid thickness (*μ*m)	Visual acuity	GCL	INL	OPL	ONL	Foveal cavity
1	8	147	387	0.2	152	207	0.3	N	Y	Y	N	N
2		214	394	0.8	377	330	1.1	N	Y	N	N	N

3	11	658	330	0.4	659	387	0.3	Y	Y	Y	N	Y
4		655	350	0.4	684	409	0.5	Y	Y	Y	N	Y

5	9	549	352	0.4	532	349	0.2	Y	Y	Y	N	Y
6		584	402	0.1	534	354	0.2	Y	Y	Y	N	Y

7	5	314	491	0.4	247	330	0.2	Y	Y	N	Y	Y
8		354	304	0.5	314	343	0.3	Y	Y	Y	N	Y

9	7	612	292	0.6	307	354	0.5	Y	Y	N	N	Y
10		520	337	0.5	300	382	0.4	Y	Y	N	N	Y

11	5	727	277	0.7	768	363	0.7	Y	Y	Y	Y	Y
12		869	301	0.7	871	319	0.8	Y	Y	Y	N	Y

Average	7.5 ± 2.3	516.9 ± 217.0	351.4 ± 60.0	0.48 ± 0.21	478.8 ± 229.1	343.9 ± 50.4	0.46 ± 0.28	83.3%	100%	66.7%	16.7%	83.3%

^*∗*^*P* value		0.622^*∗*^	0.406^*∗*^	0.323^*∗*^				10 eyes	12 eyes	8 eyes	2 eyes	10 eyes

GCL: ganglion cell layer; INL: inner nuclear layer; OPL: outer plexiform layer; ONL: outer nuclear layer.

^*∗*^*P* value: statistical comparison of two groups (the first visit versus the last visit).

**Table 2:** Correlations of clinical and optical coherence tomographic characteristics of the patients.

		Visual acuity	Foveal thickness	Choroid thickness
Visual acuity	Pearson correlation	1	0.280	−0.190
	Sig. (2-tailed)		0.185	0.373
	*N*	24	24	24

Foveal thickness	Pearson correlation	0.280	1	−0.092
	Sig. (2-tailed)	0.185		0.670
	*N*	24	24	24

Choroid thickness	Pearson correlation	−0.190	−0.092	1
	Sig. (2-tailed)	0.373	0.670	
	*N*	24	24	24
